# Structural and Functional Insights into a Novel *Aspergillus ochraceus* Polysaccharide from the Weddell Sea: Implications for Melanoma Immunotherapy In Vitro

**DOI:** 10.3390/md23060246

**Published:** 2025-06-10

**Authors:** Jiale Hao, Kouame kra Wilfred Armel, Pengcheng Gao, Jinglei Wang, Weibin Zhang, Kexin Du, Qi Li, Huishu Gao, Guangli Yu, Guoyun Li

**Affiliations:** 1 Key Laboratory of Marine Drugs, Shandong Key Laboratory of Glycoscience and Glycotherapeutics, Ministry of Education, School of Medicine and Pharmacy, Ocean University of China, Qingdao 266003, China; hjl9700@163.com (J.H.); kouamewfu@gmail.com (K.k.W.A.); gaopengchengsir@163.com (P.G.); wjinglei920@163.com (J.W.); zwb19960327@163.com (W.Z.); 15206566923@163.com (K.D.); 15166013795@163.com (Q.L.); gaohuishu0628@163.com (H.G.); 2 Laboratory for Marine Drugs and Bioproducts, Qingdao Marine Science and Technology Center, Qingdao 266237, China

**Keywords:** *Aspergillus ochraceus*, melanoma, immunotherapy, macrophages, anti-tumor, polysaccharide

## Abstract

Immunotherapy is a transformative strategy in oncology, yet the development of novel immunomodulatory agents remains essential. This study explores the anti-tumor potential of a structurally unique polysaccharide isolated from an *Aspergillus ochraceus* (AOP), sourced from the Antarctic Weddell Sea. Using alkaline-assisted extraction and chromatographic purification, we obtained a homogeneous polysaccharide predominantly composed of galactose and mannose, with an average molecular weight of 39.67 kDa. The structure was characterized by an integrated nuclear magnetic resonance spectroscopy and mass spectrometry analysis, revealing that the AOP is composed of β (1→5)-linked galactofuranose units, with a minor substitution by α-D-mannopyranose residues via (1→2) glycosidic bonds at the C2 of the galactofuranose. Functional assays, including CCK8 and wound-healing tests, demonstrated that this polysaccharide, referred to as AOP, inhibited melanoma cell proliferation and migration in a dose-dependent manner. Additionally, the AOP activated RAW264.7 and bone marrow-derived macrophage (BMDM) cells without exhibiting significant cytotoxicity, leading to the release of inflammatory factors such as TNF-α, IL-1β, and IL-6. Mechanistically, the AOP was found to upregulate the expression of CD86 and IFN-γ, while downregulating genes like IL-4 and Arg1. These findings position the AOP as the first documented Antarctic fungal polysaccharide with macrophage-reprogramming capabilities against melanoma, offering novel molecular insights for marine-derived immunotherapeutics.

## 1. Introduction

In recent years, the advent of immunotherapy has revolutionized traditional melanoma treatment approaches [1]. Increasing evidence highlights the critical role of macrophages in tumors, with their primary functions encompassing immune defense [2], immune regulation [3], and immune secretion [4]. Macrophages are adept at recognizing pathogen-associated molecular patterns (PAMPs) and damage-associated molecular patterns (DAMPs) [5]. Traditionally, activated macrophages are classified into two polarization states within the tumor microenvironment (TME), collectively known as tumor-associated macrophages (TAMs): M1-TAMs, or classically activated macrophages, and M2-TAMs, or alternatively activated macrophages [6]. M1 macrophages play a pivotal anti-tumor role by mediating phagocytosis and antibody-dependent cell cytotoxicity, while releasing pro-inflammatory cytokines such as tumor necrosis factor alpha (TNF-α), interleukin-1β (IL-1β), and interleukin-6 (IL-6) [7]. In contrast, M2-TAMs contribute to tumor development and metastasis by promoting anti-inflammatory processes, enhancing tumor angiogenesis, and facilitating tumor progression [8] through mechanisms such as the epithelial-to-mesenchymal transition (EMT) process [9]. Melanoma, known for its aggressive metastatic behavior, is significantly influenced by EMT; this process is characterized by the transition from E-cadherin to N-cadherin, along with the upregulation of genes like vimentin, slug, and snail, which enables melanoma cells to form N-cadherin-mediated adhesion with stromal fibroblasts [10]. Therefore, encouraging the conversion of M2-TAMs to M1-TAMs represents a promising therapeutic strategy, with the potential to regulate macrophage activity and overall tumor progression.

Marine ecosystems represent an underexplored resource for pharmacological compounds, with 68% of novel natural products in clinical trials derived from marine organisms, particularly deep-sea fungi that thrive under extreme conditions and have developed unique biosynthetic pathways to produce stress-adapted metabolites [11]. Genomic analyses show that deep-sea fungi contain a higher abundance of carbohydrate-active enzymes (CAZymes) compared to their terrestrial counterparts, enabling the synthesis of structurally novel glycans with distinctive sulfation patterns and branching architectures, which are inaccessible via land-based synthesis [12]. Structural diversity fosters a plethora of biological activity. The *Aspergillus ochraceus* has complex biotechnological potential. Zou found that the strain *Aspergillus ochraceus* MCCC 3A00521 isolated from the Northeastern Pacific has strong inhibitory activity against ferroptosis by downregulating HMOX-1 expression [13]; Nasr et al. discovered that a protease activator (PAPC) secreted by *Aspergillus ochraceus* can disrupt the biofilm of Staphylococcus aureus, thereby exerting antibacterial activity [14]; Guo et al. identified a galactomannan with a novel structure produced by the coral endophytic fungus *Aspergillus ochraceus*, although its biological activity remains untested [15]. Despite these findings, there is still limited research on the structure and bioactivity of cell wall polysaccharides derived from *Aspergillus ochraceus*.

In recent years, we have been focusing on exploring the anti-tumor activity of marine-derived polysaccharides. This study aims to extract and purify cell wall polysaccharides from *Aspergillus ochraceus* captured in the Weddell Sea of Antarctica, further characterize its chemical structure, and preliminarily evaluate the anti-melanoma activity in vitro. Thus, the AOP (*Aspergillus ochraceus* polysaccharide) might become a novel leading compound for melanoma treatment due to its multiple potential effects.

## 2. Results

### 2.1. Yield and Purification

The AOP (300 mg, yielding 18.2% of the crude polysaccharide) was eluted with 0.2 M NaCl from the DEAE Sepharose Fast Flow column. The polysaccharide was further purified using a Sephacryl S-200 HR column, resulting in the isolation of a polysaccharide designated AOP (200 mg, yielding 47% of *Aspergillus ochraceus* biomass).

### 2.2. Molecular Weight and Monosaccharide Composition

**Figure 1:**
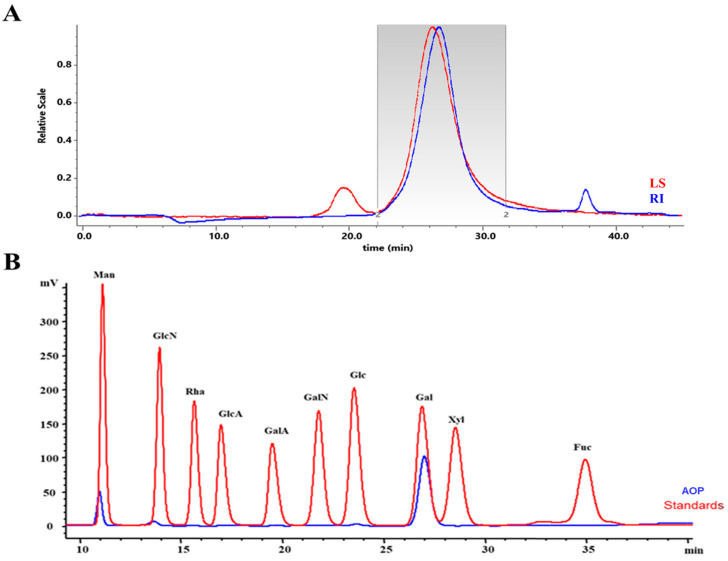
Characterization of polysaccharides. (**A**) The homogeneity of AOP using HPGPC. Peak with the molecular weight at 39.67 kDa was examined in the standard curve. (**B**) Monosaccharide composition analysis of AOP.

The HPGPC analysis displayed a single symmetrical peak at approximately 27.46 min, confirming the homogeneity of the AOP, as shown in Figure 1A. Understanding the monosaccharide composition is crucial for characterizing polysaccharides, as monosaccharides play a significant role in determining both the structure and biological activity of polysaccharides [16]. The HPLC analysis revealed that the AOP is a neutral polysaccharide (Figure 1B), primarily composed of mannose (Man) and galactose (Gal), with a molar ratio of 20.93:79.07. Notably, galactose was the predominant component in the AOP, comprising over 80% of the total polysaccharide content. This composition contrasts with findings from another study, which reported a different structure for the galactomannan produced by the coral endophytic fungus *Aspergillus ochraceus* [15]. The variations in monosaccharide composition may be attributed to differences in the extraction and purification methods employed in the studies.

### 2.3. NMR Spectroscopy Analysis

**Figure 2:**
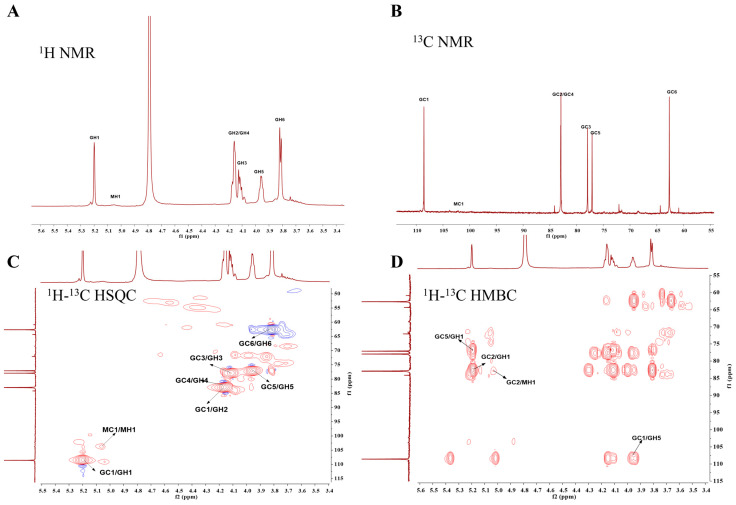
1D and 2D spectra of AOP. (**A**) ^1^H NMR spectrum; (**B**) ^13^C NMR spectrum; (**C**) ^1^H-^13^C HSQC spectrum; and (**D**) ^1^H-^13^C HMBC spectrum. Chemical shifts are expressed in ppm using acetone as an internal standard at 2.225 ppm for ^1^H and 31.07 ppm for ^13^C. G or M correspond to → 5)-β-D-Gal*_f_* (1→and Man*_p_* α (1→2)-linked to the C2 position of Gal*_f_*, respectively. Gal*_f_*: galactofuranose, Man*_p_*: mannopyranose.

The structural characterization of the polysaccharide was conducted through a comprehensive nuclear magnetic resonance (NMR) analysis. As shown in the ^1^H-NMR spectrum (Figure 2A), two distinct anomeric proton signals were observed, at 5.21 ppm (G, unsubstituted β-D-galactofuranose backbone) and 5.05 ppm (M, α-D-mannopyranose). Non-anomeric protons (H2-H6) resonated between 3.60 and 4.20 ppm. The analysis of the ^13^C-NMR spectrum (Figure 2B) revealed two characteristic anomeric carbon signals in the 90–110 ppm region: the G unit displayed an unusual C1 chemical shift at 108.48 ppm, significantly upfield-shifted compared to typical β-pyranose configurations (97–105 ppm). Our observed chemical shift data for C1 (≈108–110 ppm) corroborate prior characterizations of β-D-galactofuranose residues in *Aspergillus ochraceus* galactomannan [15,17,18,19], collectively confirming the β-furanose configuration. The corresponding anomeric proton signal (5.21 ppm) aligned with established β-Gal*_f_* H1 chemical shifts (5.0–5.5 ppm). The M unit exhibited a C1 signal at δ 102.01 ppm, within the conventional β-pyranose range. However, its associated H1 proton resonance at 5.21 ppm displayed an unusual downfield shift compared to typical β-pyranoses (4.5–5.0 ppm), suggesting α-mannopyranose configuration (5.2–5.4 ppm for α-anomers). Additional carbon signals (60.94–85.8 ppm) included a characteristic unsubstituted C6 resonance at 62.66 ppm. The ^1^H-^13^C HSQC correlation spectrum (Figure 2C) confirmed the G unit assignment through direct correlation between H1 (5.21 ppm) and C1 (108.38 ppm). The exceptional C1 chemical shift deviation from standard β-pyranoses further supported the β-Gal*_f_* assignment, suggesting potential →5)-β-Gal*_f_*-(1→ linkages through furanose ring formation. Structural connectivity was elucidated through HMBC analysis (Figure 2D). Key cross-peaks included GC5/GH1 (76.93/5.21 ppm), indicating →5)-β-Gal*_f_*-(1→ glycosidic linkages; GC1/GH5 (108.38/3.97 ppm), confirming consecutive (1→5)-linked galactofuranose residues; and GC2/MH1 (83.96/5.05 ppm), suggesting mannopyranose attachment to the C2 of galactofuranose. The quantitative analysis of ^1^H-NMR integrals revealed a G: M ratio of 14.0:1.0. This apparent discrepancy from the monosaccharide composition may arise from differential acid hydrolysis stability, as furanosidic bonds (G units) are more prone to degradation than pyranosidic linkages (M units) during acidic hydrolysis, leading to the relative enrichment of mannopyranose residues [20].

### 2.4. Mass Spectrometric Fragment Ion Analysis of AOP Oligosaccharides

**Figure 3:**
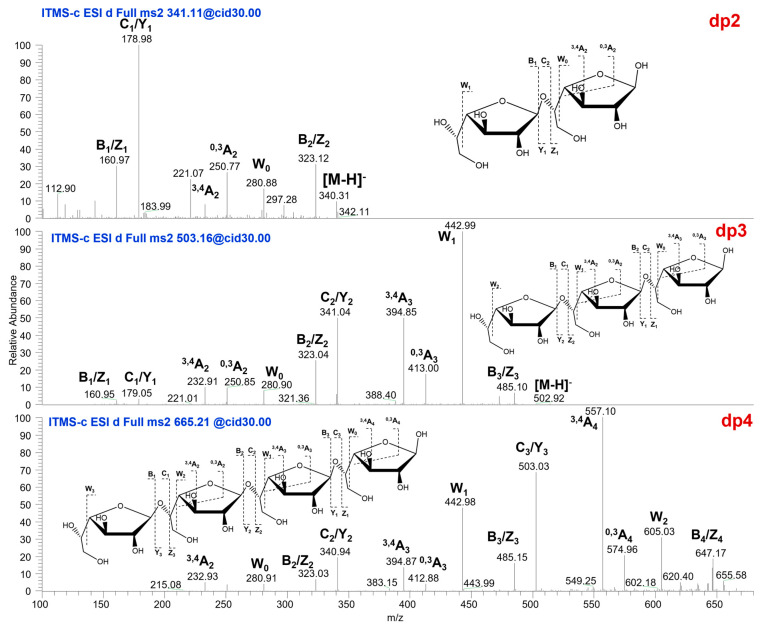
ESI-CID-MS^2^ product ion spectrum and assignments of the ions of dp2-dp4. The sequence is shown to indicate the proposed fragmentations.

The oligosaccharide fragments generated by the controlled acid hydrolysis of the AOP were characterized by ESI-CID-MS^2^. Given the structural homology with the galactomannan system reported in prior studies [15], we adopted their nomenclature system for fragment ion assignment. A typical cleavage was designated as W-series ions (W1, W2) representing C4-C5 bond cleavage at consecutive reducing-end glycosyl units, with A-type cross-ring fragments (A2, A3) assigned to non-reducing-end cleavages. A mass spectrometry analysis was conducted in negative ion mode, revealing that the controlled acid degradation products predominantly comprised dp2–dp4 oligomers. The interpretation of the oligosaccharides’ secondary fragment ions and their structural schematic diagram are illustrated in Figure 3. Characteristic C/Y-type glycosidic cleavages produced prominent ions at *m*/*z* 503.03 (Δ−162 Da) and 340.94 (Δ−324 Da), accompanied by B/Z-type fragments at *m*/*z* 323.03, 485.15, and 647.17. Cross-ring dissociation patterns included ^3,4^A-series ions (*m*/*z* 232.93, 394.87, 557.10) and ^0,3^A-type fragments (*m*/*z* 412.88, 574.96), while the W-series progression (*m*/*z* 280.91→442.98→605.03) showed sequential mass decreases of 162 Da, consistent with the loss of galactofuranose units. These spectral features were consistent with the →5)-β-Gal*_f_*-(1→ linkage pattern identified by NMR, with W-type ions confirming terminal β-Gal*_f_* positioning and ^3,4^A cleavages supporting a furanose ring geometry. The structural assignments derived from the tandem mass spectrometric analysis of MS^2^ fragment ions were systematically corroborated through a comparative evaluation with NMR spectroscopic data, demonstrating a complementary agreement in the elucidation of glycosidic linkage patterns and monosaccharide configurations. The comprehensive structural analysis reveals that the *Aspergillus ochraceus* polysaccharide (AOP) possesses a backbone composed of β-(1→5)-linked galactofuranosyl residues. This predominant furanogalactan framework is partially substituted at the C2 position of galactofuranose residues by α-D-mannopyranose units via (1→2)-glycosidic bonds. The quantitative analysis of the repeating unit demonstrates an exact stoichiometric ratio of 15 monosaccharide constituents per structural repeat unit, comprising 14 galactofuranose moieties in the main chain and 1 mannopyranose residue as the pendant group. This architectural configuration results in a distinctive polysaccharide structure characterized by periodic mannan substitutions along the galactose backbone at defined intervals.

### 2.5. Cell Proliferation Analysis

**Figure 4:**
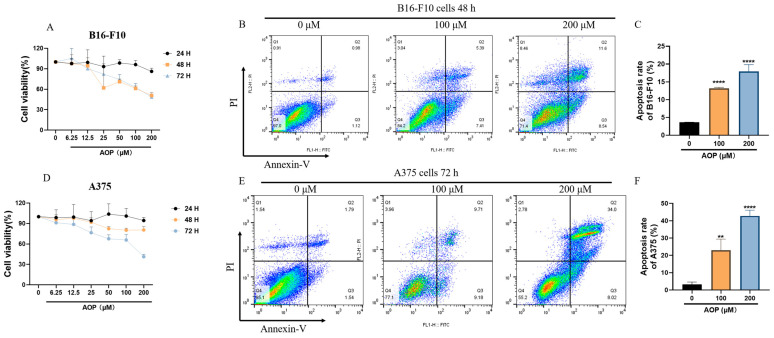
AOP could directly promote apoptosis of B16-F10 and A375 cells at high doses. B16-F10 cells (**A**) and A375 cells (**D**) were treated with different concentrations of AOP for 24, 48, and 72 h, followed by measuring cell viability by CCK8. Then, the amounts of apoptosis cells of B16-F10 cells (**B**) and A375 cells (**E**) were analyzed by flow cytometry, respectively. The statistical graph of three independent repeated experiments of (**C**,**F**). All data were obtained from three independent experiments and were presented as mean ± S.D., **, *p* < 0.01, ****, *p* < 0.0001, *n* = 3.

Melanoma is one of the most aggressive forms of skin cancer and one of the most common cancers in humans [21]. Consequently, the development of effective therapeutic drugs is urgently needed. To explore the impact of the AOP on melanoma cell proliferation, we first treated B16-F10 cells with varying concentrations of AOP. As shown in Figure 4A, the AOP treatment at 24 h exhibited no significant toxicity to B16-F10 cells. However, when the incubation time was extended to 48 and 72 h, AOP at a concentration of 50 μM significantly inhibited cell proliferation. To assess the apoptosis rate induced by the AOP, flow cytometry was employed. As illustrated in Figure 4B,C, nearly no apoptosis was observed in the control group of B16-F10 cells. After treating with the AOP at 100 and 200 μM for 48 h, apoptosis rates increased to approximately 13.16% and 17.95%, respectively. The simultaneous evaluation of AOP activity on A375 cells was performed. However, A375 cells appeared to be more sensitive to AOP treatment after 72 h, with proliferation being inhibited at lower concentrations (Figure 4D). Flow cytometry results indicated apoptosis rates of 23.02% and 42.76% at 100 and 200 μM, respectively (Figure 4E). This differential sensitivity may stem from the genetic variations between these two tumor types. Overall, it is suggested that the AOP’s primary action is to inhibit the proliferation of melanoma cells rather than to induce apoptosis.

### 2.6. AOP Might Inhibit the Migration of Melanoma Cells by Suppressing the EMT Process

**Figure 5:**
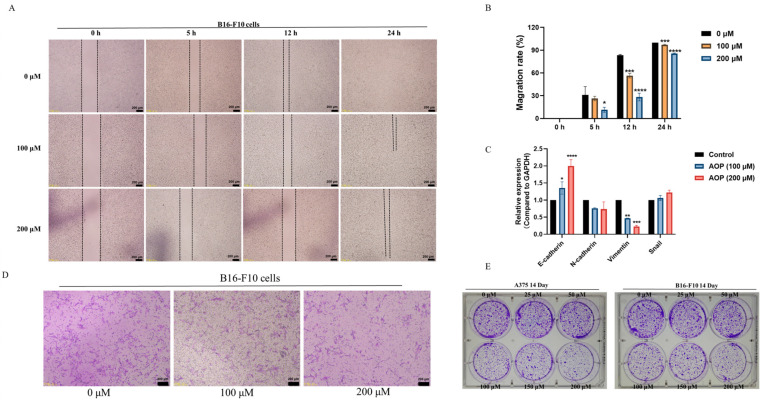
AOP could inhibit the migration of B16-F10 cells by inhibiting the EMT process. (**A**) B16-F10 cells were incubated with 100 μM or 200 μM AOP; subsequently, the wound healing of B16-F10 cells was observed at 5, 12, and 24 h, and the migration rates are shown in (**B**). Following the treatment with AOP for 12 h, total RNA was extracted and reversed transcription to cDNA, and the expression of E-cadherin, N-cadherin, snail, and vimentin were tested by qPCR assay (**C**). Furthermore, the effect of AOP on the migration ability of B16-F10 cells using transwell experiments is shown (**D**). Finally, after incubating B16-F10 cells or A375 cells with AOP or not, cultivation was continued for 14 days and the formation of clones was observed (**E**). The cell morphology was observed with Olympus CKX41 under bright field; the scale bar = 200 μm.; *, *p* < 0.05, **, *p* < 0.01, ***, *p* < 0.001, ****, *p* < 0.0001, *n* = 3.

Melanoma is recognized as one of the most metastatic cancers, presenting significant challenges for clinical treatment [22]. To investigate the effects of the AOP on the migration of B16-F10 and A375 cells, we conducted wound-healing assays, transwell migration assays, and colony formation experiments. Initially, as demonstrated in Figure 5A,B, at the 0 h mark in B16-F10 cells, the scratch widths in both the control and AOP-treated groups were comparable. However, after further incubation for 5 and 12 h, the AOP significantly inhibited wound healing; the migration rates were 56.42% and 28.21%, respectively. By 24 h, the control group had nearly completed healing, while the 200 μM AOP treatment group showed incomplete wound closure with the migration rates of 85.68%, indicating that the AOP effectively inhibits the migration of B16-F10 cells, particularly within the first 12 h.

Subsequently, as shown in Figure 5D, the AOP notably reduced the number of B16-F10 cells migrating from the upper chamber to the lower chamber of the transwell assay at concentrations of 100 and 200 μM. In the colony formation experiment, we observed a significant decrease in the number of clones formed by both B16-F10 (right) and A375 (left) cells following AOP incubation (Figure 5E). The epithelial-to-mesenchymal transition (EMT) is a critical process in cancer metastasis, during which epithelial cells acquire mesenchymal characteristics, enhancing their motility and migratory abilities [16]. Therefore, we next evaluated the expression of genes associated with the EMT process [23]. As illustrated in Figure 5C, the AOP treatment resulted in the downregulation of N-cadherin and vimentin, both of which promote EMT, while simultaneously upregulating the expression of E-cadherin. Based on these observations, we hypothesize that the AOP may inhibit the migration of B16-F10 and A375 cells by suppressing the EMT process.

### 2.7. AOP Could Exert Immune Anti-Tumor Activity In Vitro by Activating M1 Macrophages

**Figure 6:**
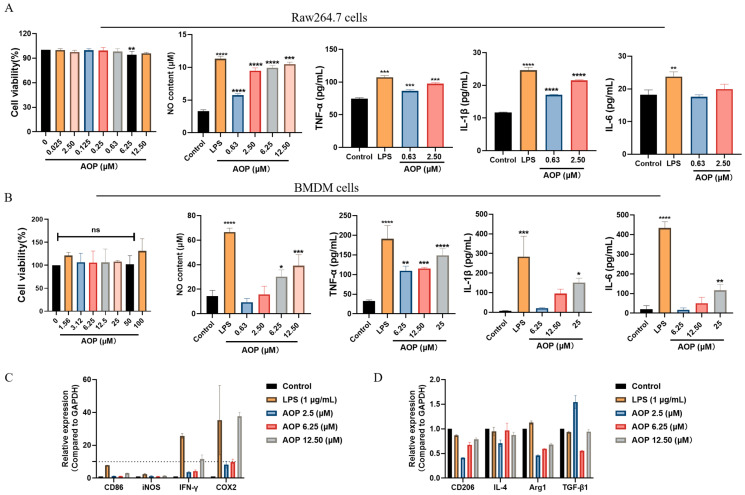
AOP could activate macrophages and promote their transformation to the M1 type at low concentrations. Raw264.7 cells (**A**) or BMDM cells (**B**) were treated with different concentrations of AOP for 24 h; then, the cell supernatant was collected, and the content of nitric oxide, TNF-α, IL-1β, and IL-6 in the supernatant was detected using ELISA assay. Total RNA of BMDM cells was extracted and reversed transcribed to cDNA, and the expression of CD86, iNOS, IFN-γ, and COX2 (**C**), and CD206, IL-4, Arg1, and TGF-β1 (**D**) was detected by qPCR assay. LPS was used as the positive control. Significance levels by two-tailed t-test: * *p* < 0.05, ** *p* < 0.01, *** *p* < 0.001, **** *p* < 0.0001.

Recent advancements in tumor immunotherapy have led to the approval of multiple immunotherapeutic drugs by the U.S. Food and Drug Administration (FDA) [24]. Macrophages play a pivotal role in anti-tumor immunity, regulating immune responses and eliminating tumor cells. However, the tumor microenvironment may “reprogram” macrophages, leading to tumor growth promotion [25]. Thus, we evaluated the effect of the AOP on macrophage activation. Our findings indicate that the AOP demonstrated no cytotoxic effects on raw264.7 cells while promoting the secretion of nitric oxide (NO), TNF-α, IL-1β, and IL-6 in a dose-dependent manner (Figure 6A). To validate these results further, we isolated primary bone marrow-derived macrophages (BMDMs), which similarly showed an enhanced release of inflammatory cytokines and NO in response to the AOP, without any signs of cytotoxicity (Figure 6B). Given the heterogeneity of macrophages in tumor settings, we hypothesize that the AOP may facilitate the reprogramming of macrophages from the M2 phenotype to the M1 phenotype. As depicted in Figure 6C,D, the AOP can downregulate M2-related genes such as IL-4, Arg1, and TGF-β1, while simultaneously upregulating M1-related genes like CD86, iNOS, IFN-γ, and COX2, supporting our hypothesis. It is worth noting that although the protein level of CD206 showed a trend of low-level increase, its expression did not exceed the baseline level of the control group. This phenomenon can be interpreted as the existence of a transitional activation state in macrophages.

**Figure 7:**
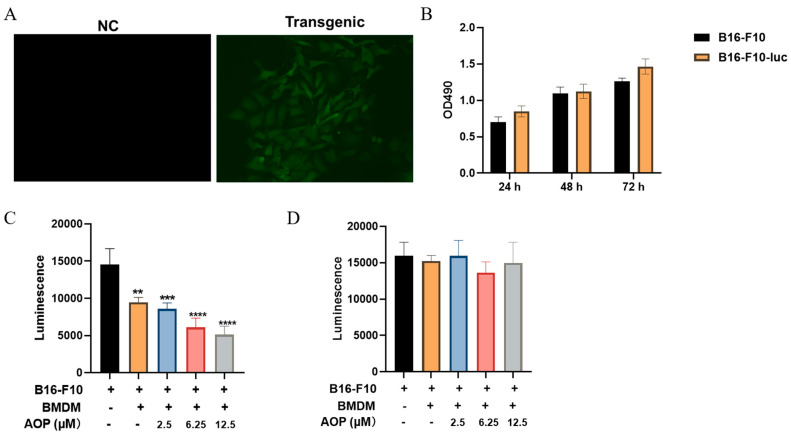
AOP could promote apoptosis of B16-F10 cells by activating BMDM at low concentrations. Briefly, plasmids containing the luc-EGFP sequence were transfected into cells to construct the B16-F10-luc stable transgenic cell line (**A**), and the difference in proliferation ability between B16-F10 and B16-F10-luc cells was evaluated (**B**). B16-F10 cells are incubated separately or together with AOP or BMDM cells for 24 hl subsequently, the proliferative ability of B16-F10 cells was evaluated using the luminescence value (**C**). The killing effect of AOP and BMDM on tumor cells without direct contact with B16-F10 was evaluated through transwell experiments (**D**). **, *p* < 0.01, ***, *p* < 0.001, ****, *p* < 0.0001, *n* = 3.

To investigate whether macrophage activation by the AOP translates into anti-tumor efficacy, we established a luciferase-expressing B16-F10 model (B16-F10-luc) for the real-time monitoring of tumor cell viability. The comparison of the transfection group to the control group revealed a strong green fluorescence, indicating successful plasmid incorporation (Figure 7A). Importantly, we noted no significant difference in proliferation between transfected B16-F10-luc cells and normal B16-F10 cells (Figure 7B). We then assessed the anti-tumor efficacy of AOP using a co-culture system through two approaches: direct contact and transwell indirect contact. In the direct contact assay, BMDM cells co-cultured with B16-F10-luc cells showed a 34.82% decrease in luminescence compared to the non-co-culture (NC) group, indicating a baseline cytotoxic effect of BMDMs. Furthermore, the AOP treatment resulted in additional reductions in luminescence, with decreases of 41.02% at 2.5 μM, 57.93% at 6.25 μM, and 64.64% compared to the NC group (Figure 7C). In contrast, when we employed the transwell indirect contact method, the anti-tumor activity of the AOP was not evident (Figure 7D). These findings suggest that the AOP can exert immune anti-tumor activity by promoting the activation of M1 macrophages, with this effect being dependent on direct cell-to-cell contact.

## 3. Discussion

The abyssal biome exerts unique evolutionary pressures that drive marine fungi to develop specialized chemobiosynthetic compensatory mechanisms [26]. While previous research on marine natural products has primarily concentrated on small-molecule secondary metabolites, the therapeutic potential of fungal structural polysaccharides remains significantly underexplored. *Aspergillus ochraceus*, a member of the *Aspergillus* genus, has been the subject of limited research regarding its polysaccharide content, particularly its cell wall polysaccharides [27]. Bardalaye et al. extracted a galactomannan peptide from the *Aspergillus niger* hyphal wall, which contains 89% of carbohydrates and 11% of proteins [28]; Gomez-Miranda, B et al. isolated alkaline soluble α-glucan and insoluble β-glucan-chitin complexes from the cell wall of *Aspergillus alliaceus* sclerotia and analyzed an α-linked extracellular polysaccharide containing *D*-galactosamine (70%), *D*-galactose (10.5%), *D*-glucose (10.5%), and acetate ions (2.6%) [29]. Guo et al. analyzed an extracellular polysaccharide, isolated from the fermented broth of the fungus *Aspergillus ochraceus* derived from coral *Dichotella gemmacea*, called AW1, which is a galactomannan containing a mannan core [15].

Contrasting with these previous investigations, which have largely focused on structural analyses, our study primarily highlights the biological activities of the cell wall polysaccharides extracted from *Aspergillus ochraceus*. We have uncovered that these polysaccharides can inhibit the proliferation of melanoma cells and activate M1 macrophages in vitro. A clear distinction emerges between the current ratio of the monosaccharide composition of the AOP and the composition of galactomannans reported. Variations in monosaccharide composition are likely to influence the structural differences, subsequently leading to diverse bioactivities related to the anti-tumor effects observed. Currently, we have conducted only in vitro activity tests. In future work, we aim to further investigate the pharmacological activities and underlying mechanisms of these polysaccharides against melanoma.

## 4. Materials and Methods

### 4.1. Chemicals and Reagents

DEAE Sepharose Fast Flow and Sephacryl S-200 HR were purchased from GE Healthcare (Danderyd, Sweden). Monosaccharide standards (galacturonic acid (GalA), glucose (Glc), galactose (Gal), xylose (Xyl), arabinose (Ara), mannose (Man), and fucose (Fuc)) were purchased from Aladdin Bio-Chem Technology Co., Ltd. (Shanghai, China). The medium potatoe dextrose water (PDA) was purchased from Hope Bio-Technology Co., Ltd. (Qingdao, China). D_2_O (99.9%) was purchased from Cambridge Isotope Laboratories, Inc. (Tewksbury, MA, USA). Puromycin (puro) and CCK8 were from Yeasen Biotechnology (Shanghai, China). Lipopolysaccharide (LPS) was obtained from Sigma-Aldrich (Saint Louis, MO, USA). Recombinant macrophage colony-stimulating factor (M-CSF) was from MedChemExpress (Shanghai, China). All other reagents used were analytical grade from Sinopharm Chemical Reagent Co., Ltd. (Shanghai, China).

### 4.2. Aspergillus Ochraceus Cultured Condition

*Aspergillus ochraceus* was deposited at the Marine Medicinal Bioresources Center (MMBC) (Ocean University of China, China), and was collected from Antarctic Weddell Sea Island and stored at the Ocean University of China’s Marine Derived Species Bank with a case number of HDN1829900. It was identified according to its morphological characteristics and 18S rRNA sequences. The marine fungus *Aspergillus ochraceus* was initially cultivated on potato dextrose water medium and grown in a Petri dish sample (One week for the growth of the strains) at 28 °C for 7 days, then inoculated into a conical flask from a total of 2 L of culture medium comprising potato dextrose water. This culture was agitated on a rotary shaker at 178 rpm and 28 °C for 9 days. Finally, the broth was then separated from the filtrate by centrifugation at 5000 rpm for 25 min to isolate the supernatant; the resulting precipitate or pellet was obtained.

### 4.3. Preparation of AOP

Autolysis of *Aspergillus ochraceus*: Freshly precipitated *Aspergillus ochraceus* cells are combined with 10 times the volume of distilled water and thoroughly mixed. The mixture is then filtered through a 100-mesh gauze to remove any impurities. The filtrate is centrifuged, and the resulting precipitate is washed three times with distilled water. The precipitate is then dried overnight in an oven at 60 °C, mixed with a 1 M sodium acetate buffer solution at a 1:5 mass ratio. The pH is adjusted to 5.5 using acetic acid, and solid sodium chloride is added to achieve a 3% mass fraction. The mixture is stirred at 55 °C for 24 h, then neutralized to pH 7 with 0.5 M sodium hydroxide solution and centrifuged. The precipitate is washed three times with distilled water, yielding *Aspergillus ochraceus* cell walls. Alkali Treatment: The precipitate is treated with a 0.5 M NaOH solution at a 1:5 mass ratio, heated, and stirred at 80 °C for 2 h. The mixture is then neutralized with HCl to a pH of 7 and centrifuged. The supernatant is concentrated under vacuum and dialyzed. The dialysate is further concentrated under vacuum and freeze-dried. Subsequently, an alkaline extract was fractionated on a DEAE Sepharose Fast Flow column (50 × 5 cm, Cl^−^) with a gradient elution of 0.2 M NaCl, and was further purified with Sephacryl S-200 HR (100 cm × 1.6 cm) column to obtain a homogeneous fraction AOP.

### 4.4. Monosaccharide Composition

The monosaccharide composition was determined using a modified 1-phenyl-3-methyl-5-pyrazolone (PMP)-HPLC method. Each marine fungi strain was hydrolyzed briefly with 2 M trifluoroacetic acid (TFA) at 110 °C for 6 h. The hydrolysate was then dried to remove the excess TFA. Next, the hydrolysate or monosaccharide standard was separately dissolved in water, followed by the addition of sodium hydroxide and PMP in methanol. The mixture was incubated at 70 °C for 1 h. After cooling, the reaction mixture was neutralized with hydrochloric acid and extracted with chloroform three times. HPLC analysis was performed on an Agilent ZORBAX Eclipse XDB-C18 column at a constant flow rate of 1.0 mL/min, with the column temperature maintained at 30 °C. The mobile phase comprised 17% acetonitrile in a 0.1 M phosphate buffer solution. The entire process was monitored using a Diode array detector.

### 4.5. NMR Analysis

The dried sample (30 mg) was dissolved with D_2_O (500 μL, 99.8% D), and 0.2 μL with acetone as an internal standard (2.225 ppm for ^1^H and 31.07 ppm for ^13^C). Then, a Bruker AVANCE III NMR spectrometer (Billerica, GermanyUSA), operating at 500 MHz at 333 K, was used to detect the NMR spectra (^1^H NMR, ^13^C NMR spectra, ^1^H-^13^C HMBC and HSQC) of the sample.

### 4.6. Mass Spectrometry

To elucidate the structural features of AOP, controlled acidic hydrolysis was performed under optimized conditions. Briefly, 10 mg/mL of AOP was dissolved in a 0.05 M H_2_SO_4_ aqueous solution and incubated at 80 °C for 1 h. The reaction was immediately quenched by neutralization with 0.1 M Ba(OH)_2_. The centrifuged hydrolysate was diluted with an equal volume of acetonitrile (1:1, *v*/*v*) prior to ESI-MS/MS analysis by LTQ Orbitrap XL (Thermo, Waltham, MA, USA). Fragment ion profiles were acquired using electrospray ionization-tandem mass spectrometry (ESI-MS/MS) in negative ion mode, with the collision-induced dissociation (CID) energy set to 20–35 eV to optimize the spectral resolution of glycosidic bond cleavage patterns. The mass data were analyzed by Xcalibur 4.0.

### 4.7. Cell Lines and Extraction of Primary BMDM Cells

B16-F10 cells were cultured in Roswell Park Memorial Institute (RPMI-1640), containing 10% fetal bovine serum (FBS, Gibco, Grand Island, NY, USA) and 1% penicillin/streptomycin; A357 cells were maintained with Dulbecco’s modified Eagle’s medium (DMEM) containing 10% (FBS, Gibco, USA) and 1% penicillin/streptomycin.

Briefly, C57BL/6 mouse bone marrow (C57BL/6 mice were purchased from GemPharmatech (Nanjing, China)) was washed to obtain a single-cell suspension, followed by steps such as red blood cell lysis and washing, and then stimulated with M-CSF (20 ng/mL) for 5 days to differentiate into BMDM, and cultured with DMEM. All cells were cultured at 37 °C in a humidified incubator with 5% CO_2_.

### 4.8. Cell Proliferation and Apoptosis Detection

The cell proliferation and apoptosis of B16-F10 and A375 cells were tested by CCK8 assay, flow cytometry assay, and clone formation assay. Briefly, cells were seeded into a 96-well plate (4 × 10^3^ cells/well) and treated with different concentrations of AOP for the intended time; then, 10 μL CCK8 was added to each well, and the optical density was measured by a spectrophotometer (Epoch 2, BioTe, Irving, TX, USA) at 450 nm. On the other hand, cells were plated into the 6-well plate (2 × 10^5^ cells/well); then, AOP was added to the cells after cells adhered, and the cell apoptosis rate was tested by Annexin V-FITC Apoptosis Detection Kit (Beyotime, Shanghai, China), following the instructions. Subsequently, flow cytometry (Novocyte 3000, Agilent, Santa Clara, CA, USA) was used for detection, and data analysis was conducted using software NovoExpress (Version 1.5.8).

As for the clone formation assay, B16-F10 cells were incoluted into a 6-well plate (600 cells/well) and continued to culture for 14 days. The medium containing AOP (or not) was changed every 3 days and the cell status was observed; after cloning, washing with PBS was performed. Then, 1 mL of 4% paraformaldehyde (Servicebio, Wuhan, China) was added to each well for 15 min and washed once with PBS; 1 mL of crystal violet staining solution (Servicebio, Wuhan, China) was added to each well and incubated for 20 min; Cells were then washed several times with PBS and photos were taken with a digital camera.

### 4.9. Cell Migration Ability Detection

The migration ability of cells was tested by wound-healing experiments and transwell experiments. Cells were plated into 6-well plate (1 × 10^6^ cells/well); after the cells are fully attached to the wall, a 10 μL pipette tip was used to draw a straight line that intersected with the marked line, and gentle rinsing with PBS buffer was performed to remove the scratched cells; culture medium containing AOP or not was added, and an Olympus CKX41 microscope (Olympus, Tokyo, Japan) was used to observe the healing of the wound. Finally, an edge analysis was conducted using ImageJ software 2.9 (NIH, Bethesda, MD, USA).

A transwell with an aperture of 8 μm was purchased from Corning (Corning, New York, NY, USA). Generally, the cells were pre-starved for 4 h, and 600 μL of culture medium containing 10% FBS was added to the lower chamber of a 24-well plate. Then, 100 μL of cell suspension (5 × 10^4^) was added to the upper chamber of the transwell, significantly, with or without AOP in both chambers. Incubation was continued for 24 h, followed by crystal violet staining as described in 4.7, and observation under a microscope.

### 4.10. RT-qPCR Assay

Cells were treated with different concentrations of AOP, total RNA was extracted by Tissue/Cell Fast RNA Extraction Kit for Animal (Abclonal, Wuhan, China), and then ABScript III RT Master Mix for qPCR with gDNA Remover (Abclonal, Wuhan, China) was used for the reverse transcription of RNA and qPCR assay. Finally, a thermocycler (Bio-Rad, Hercules, CA, USA) was used for the qPCR test. Primers (Table 1) were synthesized by Sangon Biotech (Shanghai, China).

### 4.11. ELISA Assay

Raw264.7 and BMDM cells were seeded into a 24-well plate and treated with different concentrations of AOP for 24 h. Then, the supernatant was collected and centrifugation at 13,000 rpm at 4 °C for 15 min. The contents of TNF-α, IL-1β, and IL-6 were detected using the ELISA kit (Thermo Fisher Scientific, Waltham, MA, USA) according to the manufacturer’s instructions.

### 4.12. Tumor and Immune Cell Co-Incubation Assay

A B16-F10-luc stable cell line was constructed through the lentiviral infection method. Briefly, plasmids containing the luc-EGFP sequence were transfected into cells to construct the stable transgenic cell line with a 1 week screening of puromycin, and then the killing effect was evaluated under a co-incubation system through direct contact and transwell (0.4 μm) indirect contact. After incubation with AOP-trained or untrained BMDMs, luciferase substrate was added, and the luminescence value was measured to evaluate tumor cell viability.

### 4.13. Statistics Assay

Data were displayed as mean ± S.D. and analyzed by GraphPad Prism 9.0 software using an unpaired *t*-test and one-way ANOVA. All experiments were conducted independently at least 3 times and *p* values less than 0.05 were considered significant differences (*, *p* < 0.05; **, *p* < 0.01; ***, *p* < 0.001, ****, *p* < 0.0001).

## 5. Conclusions

In conclusion, a novel homogeneous polysaccharide, designated as AOP, was successfully extracted and purified from *Aspergillus ochraceus*. Our studies demonstrated that the AOP directly inhibits the proliferation and migration of B16-F10 and A375 cells, inducing apoptosis at higher concentrations, while also exerting immune anti-tumor activity by activating macrophages at lower concentrations. These findings provide a strong rationale for further in vivo experiments. Mechanistic investigations indicated that the inhibition of migration induced by AOP may be linked to its ability to suppress the activation of the epithelial-mesenchymal transition (EMT) process. Therefore, our results lay a theoretical foundation for the development of anti-tumor lead compounds derived from marine fungi.

## Figures and Tables

**Table 1:** RT-qPCR primer sequences.

Target Gene	Forward Primer	Reverse Primer
*E-cadherin*	CAGTTCCGAGGTCTACACCTT	TGAATCGGGAGTCTTCCGAAAA
*N-cadherin*	AGGCTTCTGGTGAAATTGCAT	GTCCACCTTGAAATCTGCTGG
*vimentin*	CGTCCACACGCACCTACAG	GGGGGATGAGGAATAGAGGCT
*snail*	CACACGCTGCCTTGTGTCT	GGTCAGCAAAAGCACGGTT
*GAPDH*	AGGTCGGTGTGAACGGATTTG	GGGGTCGTTGATGGCAACA
*CD86*	TCAATGGGACTGCATATCTGCC	GCCAAAATACTACCAGCTCACT
*CD206*	CTCTGTTCAGCTATTGGACGC	TGGCACTCCCAAACATAATTTGA
*iNOS*	GTTCTCAGCCCAACAATACAAGA	GTGGACGGGTCGATGTCAC
*IFN-γ*	GACAACTACACCCTAAAGTGGAG	GCTCTGACACGAAACTGTGTTTT
*COX-2*	TTCCAATCCATGTCAAAACCGT	AGTCCGGGTACAGTCACACTT
*IL-4*	GGTCTCAACCCCCAGCTAGT	GCCGATGATCTCTCTCAAGTGAT
*Arg1*	CTCCAAGCCAAAGTCCTTAGAG	GGAGCTGTCATTAGGGACATCA
*TGF-β1*	CCACCTGCAAGACCATCGAC	CTGGCGAGCCTTAGTTTGGAC

## Data Availability

Data will be made available on request.

## Abbreviations

The following abbreviations are used in this manuscript:

BMDM	bone marrow-derived macrophage
TNF-α	Tumor necrosis factor alpha
IL-6	Interleukin-6hree letter acronym
DAMPs	Damage-associated molecular patterns
IL-1β	Interleukin-1β
TAMs	Tumor-associated macrophages
EMT	Epithelial-to-mesenchymal transition
